# Magnetic Nanoparticle Assisted Self-assembly of Cell Penetrating Peptides-Oligonucleotides Complexes for Gene Delivery

**DOI:** 10.1038/s41598-017-09803-z

**Published:** 2017-08-22

**Authors:** Moataz Dowaidar, Hani Nasser Abdelhamid, Mattias Hällbrink, Krista Freimann, Kaido Kurrikoff, Xiaodong Zou, Ülo Langel

**Affiliations:** 1 Department of Neurochemistry, Stockholm University, Svante Arrhenius väg 16B, Stockholm, SE-10691 Sweden; 2 Department of Materials and Environmental Chemistry, Stockholm University, Svante Arrhenius väg 16C, Stockholm, SE-106 91 Sweden; 3 Laboratory of Molecular Biotechnology, Institute of Technology, University of Tartu, Nooruse, Tartu 50411 Estonia

## Abstract

Magnetic nanoparticles (MNPs, Fe_3_O_4_) incorporated into the complexes of cell penetrating peptides (CPPs)-oligonucleotides (ONs) promoted the cell transfection for plasmid transfection, splice correction, and gene silencing efficiencies. Six types of cell penetrating peptides (CPPs; **P**ept**F**ect220 (denoted PF220), PF221, PF222, PF223, PF224 and PF14) and three types of gene therapeutic agents (plasmid (pGL3), **s**plicing **c**orrecting **o**ligonucleotides (SCO), and **s**mall **i**nterfering RNA (siRNA) were investigated. Magnetic nanoparticles incorporated into the complexes of CPPs-pGL3, CPPs-SCO, and CPPs-siRNA showed high cell biocompatibility and efficiently transfected the investigated cells with pGL3, SCO, and siRNA, respectively. Gene transfer vectors formed among PF14, SCO, and MNPs (PF14-SCO-MNPs) showed a superior transfection efficiency (up to 4-fold) compared to the noncovalent PF14-SCO complex, which was previously reported with a higher efficiency compared to commercial vector called Lipofectamine™2000. The high transfection efficiency of the new complexes (CPPs-SCO-MNPs) may be attributed to the morphology, low cytotoxicity, and the synergistic effect of MNPs and CPPs. PF14-pDNA-MNPs is an efficient complex for *in vivo* gene delivery upon systemic administration. The conjugation of CPPs-ONs with inorganic magnetic nanoparticles (Fe_3_O_4_) may open new venues for selective and efficient gene therapy.

## Introduction

The last decades were a witness for the advances of the technologies of disease treatment based on nucleic acid (NA) or oligonucleotides (ONs) for manipulation of gene expression, functions, regulations, and therapies^1–5^. However, cell transfection using ONs showed low cell transfection efficiency due to the poor permeability of the plasma membrane and relatively rapid degradation rate of ONs^6^. Furthermore, **s**mall **i**nterfering **r**ibonucleic **a**cid (siRNA), which causes silencing of gene expression in a sequence-specific manner and thus disrupts specific molecular pathways in various diseases^7^, suffers from high negative charges (∼40 negative charges) and high molecular weight (∼14 kDa) that prevent cell internalization^8^. These drawbacks limited the development of oligonucleotide-based therapeutic treatments. Thus, a large number of non-viral strategies were reported for the delivery of ONs^1–5^.

Various types of nanoparticles including magnetic nanoparticles (MNPs) offer exciting opportunities for gene or drug delivery^9^. Among several thousand types of inorganic nanoparticles, drug or gene delivery using magnetic nanoparticles is promising due to their high efficiency for the drug delivery, simple modification of its surface and the material biocompatibility^10–18^. The association of MNPs with gene vectors was defined as magnetofection, enhanced gene transfer especially in the presence of a magnetic field^19^. The surface of MNPs can be easily tailored using a vast number of biomolecules^20^. The selective targeting of drug delivery systems using MNPs can be improved via biomolecule recognition or magnetic targeting^21^. Peptide-conjugated iron oxide nanoparticles (Fe_3_O_4_) showed higher efficiency after activation using locally convert alternating magnetic field (AMF)^22^. MNPs provide a targeted gene delivery, protect the gene therapeutic agents against nuclease degradation and improve their stability^23–25^. Functionalization of iron oxide nanoparticle (γFe_2_O_3_ NPs) surface with phosphorothioate oligonucleotide (ODN) (Li28) and cationic peptide (Arg_15_), to form the complex of γFe_2_O_3_@PolyR/Li28/Arg_15_ was reported^26^. The positively charged surfaces of γFe_2_O_3_ NPs assisted the complexation of the NPs with the negatively charged phosphorothioate groups (pK_a_ ~ 1–2) at pH = 2.5 via electrostatic interactions^26^.

Among various strategies, cell penetrating peptides (CPPs, cationic and amphipathic peptides up to 30 amino acids in length) offered a promising platform for the delivery of ONs as non-viral vectors^27, 28^. CPPs showed high ability to form stable noncovalent complexes with ONs such as **s**plice **c**orrecting **o**ligonucleotides (SCO), siRNA and plasmids^29, 30^. CPPs provide very promising tools for delivery of gene therapeutic agents, and possess the ability to translocate across cellular plasma membranes^27^. They contain a vast number of peptides with different length and functional groups^27^. CPPs showed remarkable efficiency for nucleic acid delivery^31^. They are simple in theory but complex in practice and require further improvements.

Magnetic nanoparticles (MNPs, Fe_3_O_4_ with an average particle size 6.4 nm) modified with CPPs-ONs complex were synthesized, characterized and applied for gene therapy. Six different types of CPPs, combined with three types of gene therapeutic agents (plasmid (pGL3), SCO, and siRNA), were tested. The materials were synthesized and characterized using powder X-ray diffraction (XRD), transmission electron microscopy (TEM), scanning electron microscopy (SEM), energy dispersive X-ray spectroscopy (EDX), and fluorescence spectroscopy. Data confirmed the successful conjugation of MNPs with CPPs-ONs complexes. Fe_3_O_4_ MNPs and the complexes show high biocompatibility. Compared to the noncovalent complexes of CPPs-SCO or CPPs-pGL3, magnetic nanoparticles conjugated with CPPs-SCO or CPPs-pGL3 showed supreme activity. For instance, PF14-SCO-MNPs showed superior transfection efficiency (up to 4-fold) compared to the PF14-SCO complex, which was previously reported with a higher efficiency compared to commercial vector called Lipofectamine™2000^32^.

## Materials and Methods

FeSO_4_, Fe_2_(SO_4_)_3_ **·** *n*H_2_O, ammonium hydroxide solution (NH_4_OH), methanol, sodium bicarbonate (NaHCO_3_), trypsin (0.25%) and fetal bovine serum (FBS) were obtained from Sigma-Aldrich (Germany). Luciferase expressing plasmid (pGL3, 3 MDa, Promega, Sweden), SCO (5′-CCU CUU ACC UCA GUU ACA, 6.5 kDa), or siRNA (5′-GGA CGA GGA CGA GCA CUU CUU, 13 kDa, Microsynth AG, Switzerland) were purchased and used without purification.

All animal experiments and procedures were carried out in Estonia, which were approved by the Estonian Laboratory Animal Ethics Committee (approval no. 81, dated 04.04.2016) following relevant guidelines and regulations.

### Synthesis of magnetic nanoparticles (Fe_3_O_4_, MNPs)

Magnetic iron oxide nanoparticles (Fe_3_O_4_, MNPs), with an average diameter of 6.4 nm, was prepared using the hydrothermal co-precipitation method with modification^33^. Briefly, 0.63 g of FeSO_4_ and 1.73 g of Fe_2_(SO_4_)_3_ **·** *n*H_2_O were dissolved in deionized water (25 mL). Then, 40 mL of ammonium hydroxide (NH_4_OH) solution (28%) was added rapidly. The reaction mixture was stirred at 90 °C under reflux for 3 h. The product was washed with water (3 × 20 mL) and ethanol (95%, 2 × 20 mL), and collected using centrifugation (13500 rpm, 30 min).

### Peptide synthesis

Six different types of cell penetrating peptides (CPPs), called PF220 (Stearyl- KWLKLWFLKLLKKFL-amide), PF221 (Stearyl-FLKLLKKFLFLKLLKKFL-amide), PF222 (Stearyl-WLRLLWKKWFLKL-amide), PF223 (Stearyl-KWLKLWAGYLLGKINL-amide), PF224 (Stearyl-WKKWINLKALINLKAL-amide)^34^ and PF14 (Stearyl-AGYLLGKLLOOLAAAALOOLL-NH_2_)^32^ were tested. All the peptides (PF220, PF221, PF222, PF223, PF224, and PF14) were synthesized on an SYRO II peptide synthesizer (MultiSynTech, Germany), using fluorenyl methyloxycarbonyl chemistry applying Rink-amide Chem matrix resin (PCAS BioMatrix, Canada).

### Synthesis of peptide–plasmid complexes-magnetic nanoparticles (MNPs)

**Figure 1:**
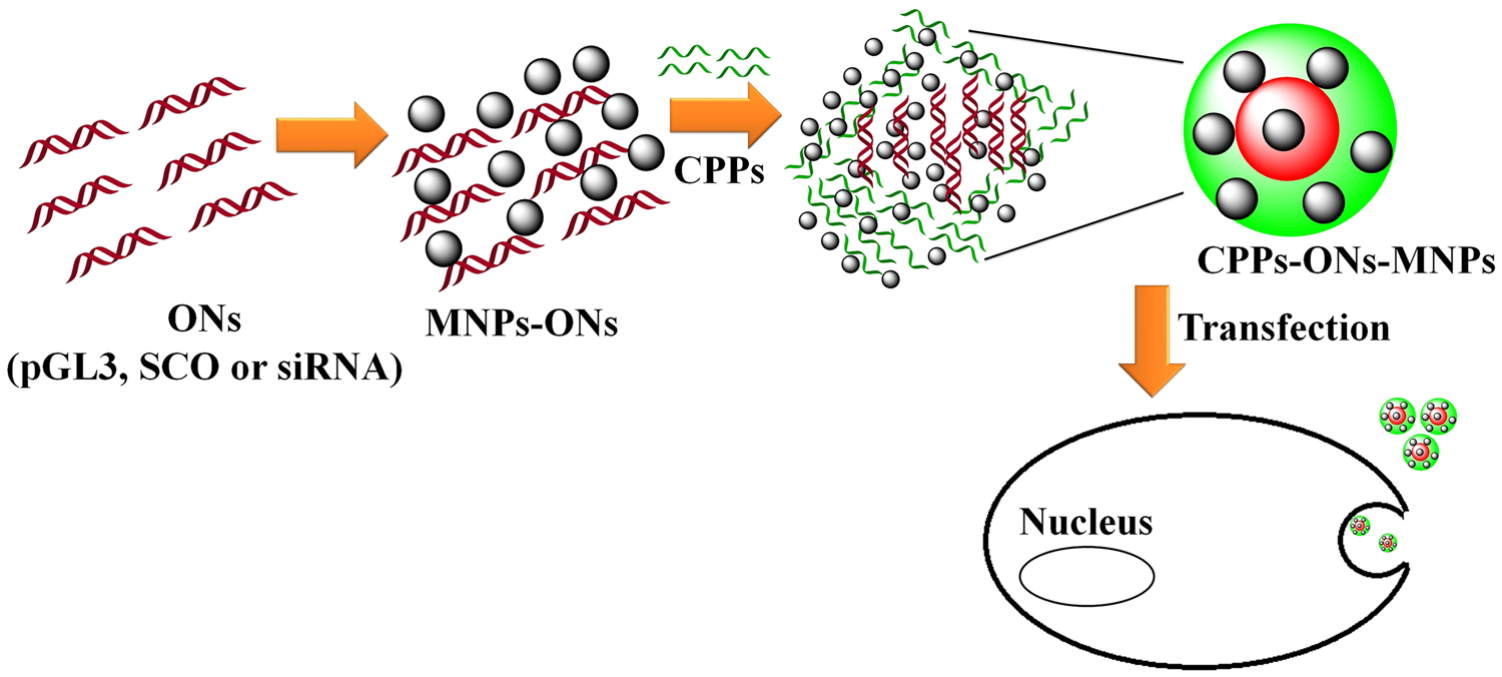
Schematic representation of CPP-ONs-MNPs noncovalent complexes. The size of the objects is not considered.

Peptide–plasmid complexes without magnetic nanoparticles were formed in Mili-Q water (MQ, 18.2 MΩ **·** cm at 25 °C) as described in our previous report^34^. Peptide–plasmid complexes modified with magnetic nanoparticles were formulated using a charge ratio (CR) of 5:1 (CPPs:pGL3) for the pGL3, a molar ratio (MR) of 10:1 (CPPs:SCO) for SCO (10 μM) and a molar ratio 20:1 for siRNA (50 and 25 nM). A suspension of magnetic nanoparticles (1 mg/mL) was prepared using MQ water. In a small vial, 80 μL of MQ water, 4 μL of MNPs (1 mg/mL), and 4 μL of pGL3 (205 ng/μL) were mixed. Finally CPPs (PF220, PF221, PF222, PF223, PF224, or PF14, 10.5 μL) were added and left at room temperature for 2 h (Fig. 1).

For SCO and siRNA, MQ water (80 μL), 4 μL of MNPs (1 mg/mL), 10.5 μL of SCO (10 μM) or siRNA (25 or 50 nM), and finally CPPs (PF220, PF221, PF222, PF223, PF224 and PF14, 10.5 μL) were added in this sequence and left at room temperature for 2 h. siRNA of 25 nM in concentration was prepared using 6 μL of siRNA solution.

### Cell culturing and treatment

Hela cells (7000 cells of 100 μL per well, 96 well plates) were cultured and used as a model for our study. Hela cells were cultured overnight in Dulbecco’s modified Eagles medium (DMEM) containing 1.0 mM sodium pyruvate, 1.0 mM glutamate, 0.1 mM non-essential amino acids, 100 mg/mL streptomycin (Invitrogen, Sweden), 10% FBS, with 100 U/mL penicillin.

HeLa puLc 705 cells were kindly provided by Prof. R. Kole’s lab. The U-87 MG-luc2 cells and HeLa puLc 705 cells were grown at 37 °C, 5% CO_2_ in DMEM with glutamic supplemented with 0.1 mM non-essential amino acids, 10% FBS, 200 U/mL penicillin, and 200 mg/mL streptomycin (Invitrogen, Sweden).

### The effect of MNPs on the fluorescence signal of Alexa Fluor 568 oligonucleotide amine

The fluorescence signal upon the interaction of Alexa Fluor 568 oligonucleotide with MNPs was measured. The kits have been optimized for labeling reactions with 50 µg of 5′-amine—modified oligonucleotide (18 to 24 bases in length).

### Water-soluble tetrazolium salt-1 (WST-1) toxicity assay

Cytotoxicity of the materials was measured using cell proliferation reagent (WST-1, Roche Diagnostics Scandinavia AB, Sweden). Hela cells (7000 cells/well) were seeded in 96-well plates, and after 24 h the cells were treated with peptide–plasmid complexes modified magnetic nanoparticles. The wells containing the treated cells were incubated for another 24 h. The cytotoxicity was measured by following the absorbance at 450 nm on Sunrise™-Tecan microplate absorbance reader.

### Determination of the efficacy of complex formation

#### Expression of pGL3 luciferase plasmid

The efficiency of CPPs-pGL3-MNPs was evaluated using Hela cells (7000 cells per 100 μL per well in 96 well plate). The activity was measured using pGL3 luciferase expressing plasmid (Promega, USA) using Qiagen Plasmid Midi kit (Qiagen, USA). Typically, 10 μL of CPPs-pGL3-MNPs (CPPs: PF220, PF221, PF222, PF223, PF224, and PF14) was added to Hela cells (7000 cells/100 μL). Then, the cells were incubated for 24 h. After incubation, the medium was decanted, and the cells were lysed in cell lysis buffer (Promega, USA). Finally, luciferase activity was measured by using GLOMAX™ luminometer (Promega, USA).

#### Splice correction oligonucleotides assay (SCO)

The biological activity of CPPs-SCO-MNPs (CPPs: PF220, PF221, PF222, PF223, PF224, and PF14) was determined using HeLa puLc 705 cells (7000 cells/100 μL)^35^. The media of cell were aspirated from each well and followed by the addition of 10 μL of the lysis solution (0.2% Triton X-100 in HKR buffer). Finally, luciferase activity was measured using Promega’s luciferase assay system on GLOMAX™ 96 microplate luminometer (Promega, Sweden). The complex was also separated using external magnets (after 5 min) and redispersed in water (1 mL) before the assessment of biological activity.

#### Transfection of siRNA

siRNA and PF14 were mixed in a molar ratio of 1:20 with and without magnetic nanoparticles. U-87 MG-luc2 cells (7000 cells/100 μL) were cultured and investigated for transfection of siRNA. After the cells have been treated with PF14-siRNA or PF14-siRNA-MNPs (10 μL) for 24 h, the plates were handled using the same procedure as that for the SCO experiments described above.

#### Scavenger receptor A (SCARA) inhibition

For SCARA inhibition experiments, antagonists (polyinosinic acid (poly I, 10 μg/mL, 671.4 g/mol), dextran sulphate (Dex, 2.5 μg/mL, 40 kDa) and fucoidan (Fuc, 2.5 μg/mL, 20–200 kDa)) and controls (polycytidylic acid (poly C, 10 μg/mL, 671.1 g/mol), chondroitin sulphate (Chon, 2.5 μg/mL, 463.4 g/mol) and galactose (Gal, 2.5 μg/mL, 180.2 g/mol)) were investigated using the above concentrations 1 h prior to the addition of PF14-SCO-MNPs. Hela 705 cell lines were treated for 1 h with an inhibitor or a control. Then, 10 μL of the previously described complex PF14-SCO or PF14-SCO-MNPs was added. Splice correction was measured after 24 h by measuring the luminescence signals using GLOMAX™ 96 microplate luminometer (Promega, Sweden). The signal values were normalized to the highest activity of PF14-SCO.

### *In vivo* study of PF14-plasmid DNA (pDNA) with and without MNPs

50 µg of Fe_3_O_4_ was first mixed with 20 µg of pDNA, then with PF14 at CR4 in 150 µL of MQ (the dose for one single animal) and incubated at room temperature for 1 h before injection. The injection was performed in 200 µL of 5% glucose, which was added immediately before the injection. The complexes were injected i.v. (via tail vein) using Hsd:Athymic Nude-Foxn1nu female mice (4–6 week old, Harlan, UK, group sizes 3–5). Gene expression levels were evaluated post-mortem 24 hours after the injection.

The whole tissues were homogenized using a Precellys®24-Dual homogenization system (Bertin Technologies, France) and lysed using 1 × Promega lysis buffer (Promega, Sweden). To analyze the content of luciferase, homogenized tissues were thawed and 500 µL of Promega Reporter lysis 1× buffer was added. The sample was subsequently vortexed for 15 minutes, subjected to three consecutive freeze-thaw cycles (liquid nitrogen and 37 °C water bath), centrifuged for three minutes at 10 000 G, 4 °C; the supernatant was removed and saved for further analysis. 500 µL of lysis buffer was again added to the pellet and the extraction process repeated (without freeze-thaw cycles). The second supernatant was combined with the first one and subjected to luciferase activity assay. Luciferase was measured with Promega luciferase assay system, in combination with GLOMAX 96 microplate luminometer (Promega, Sweden). For this, 20 µL of the supernatant was transferred to a white 96-well plate and 80 µL of the luciferase substrate was added to each sample.

An average LU (light unit) out of three technical replicates was used for data analysis. The average LU values within each sample were normalized to the protein content (BioRad, United States). The resulting RLU/mg was further normalized to the corresponding tissue of the animal that received the naked pDNA injection (sham treatment).

### Statistical analysis

Data were generated from at least three independent experiments and statistically analyzed using two-way ANOVA test (the program of Graphpad Prism) for the statistical significance: *p < 0.05; **p < 0.01; ***p < 0.001; ****p < 0.0001.

### Instrumentation and sample preparation

Transmission electron microscopy (TEM) images and energy dispersive X-ray analysis (EDX) were recorded using JEM-2000FX (JEOL, Japan) at accelerating voltage 200 kV. Drops of the material suspension were transferred to a carbon-coated Cu grid (3 mm) on a filter paper, which was dried using a Lamb 100 W for 30 min. Scanning electron microscopy (SEM) images and EDX were recorded using JSM-7000 (JEOL, Japan) at accelerating voltage 15 kV. The material suspension was dropped on a carbon-coated holder and dried as described for TEM. X-ray diffraction (XRD) of the bare magnetic nanoparticles was recorded using a PANalytical X’Pert diffractometer (Germany, accelerating voltage 45 mV, current 40 mA). Zeta potentials were estimated at 25 °C using Malvern Zetasizer Nano ZS particle analyzer (Malvern Instruments Ltd., Malvern) at a wavelength of 532 nm with a solid-state He–Ne laser at a scattering angle of 173°.

### Confocal microscopy

HeLa705-cells (7000) were seeded in 96-well plate with glass bottom 24 h before the treatment. The cells were incubated in serum-containing media containing PF14-Alexa 568-705ASO (MR10, 100 nM of SCO) complexes for 24 h, and then treated with Fast-dio membrane stain according to the manufacturer’s instruction. Imaging was performed using a Leica DM/IRBE 2 epi-fluorescence microscope controlled using Micro-Manager^Ref^ with a 63 × 1.4 NA oil immersion objective. Images were analyzed using Fiji (ImageJ) software. Image stacks were filtered with the 3d mean filter using 2 × 2 × 2 setting.

## Results and Discussion

### Characterization of iron oxide (Fe_3_O_4_) magnetic nanoparticles (MNPs)

**Figure 2:**
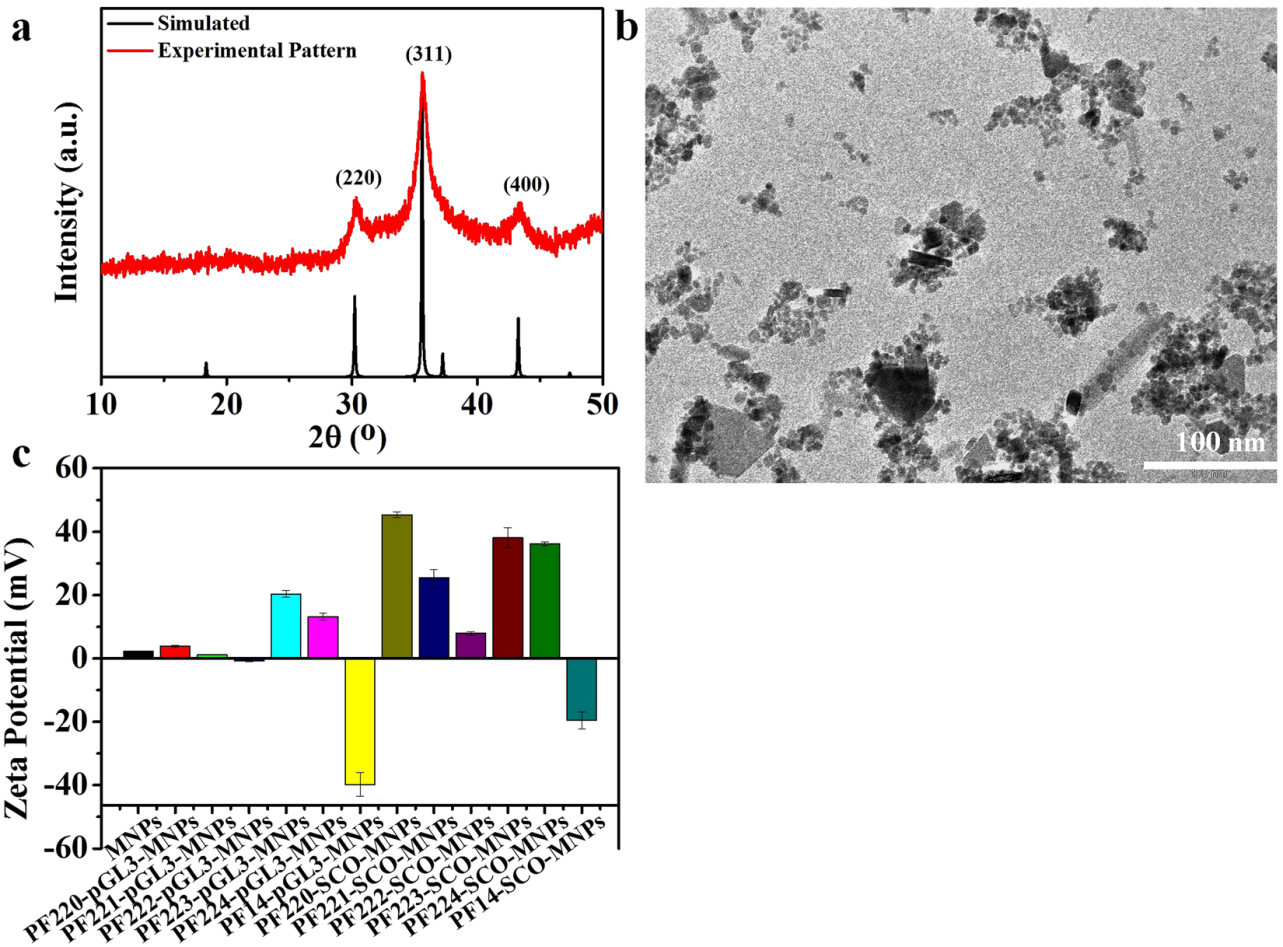
Characterization of MNPs using (**a**) XRD, (**b**) TEM, and (**c**) zeta potential.

The experimental X-ray diffraction pattern of the MNPs is in good agreement with the simulated X-ray diffraction pattern of Fe_3_O_4_ (ICSD-250540, space group *Fd*-3*m*, *a* = 8.358 Å, Fig. 2a)^36^. As shown from the TEM images, most MNPs are less than 10 nm (Fig. 2b), with a narrow size distribution and an average particle size of about 6.4 nm (Figure S1). MNPs of less than 10 nm in sizes show superparamagnetic properties (**s**uper **p**aramagnetic **i**ron **o**xide (SPIO)^9, 37^. The zeta potential of the dispersed MNPs was 2.2 ± 0.1 mV (Fig. 1c). This value confirms the low stability of the MNPs in dispersion and their tendency to aggregate. SEM images show the aggregation of the small magnetic nanoparticles (Figure S2).

### Characterization of CPPs and CPPs-gene therapeutic agents (pGL3, SCO, siRNA) modified using MNPs

Six different types of cell penetrating peptides (CPPs); namely PF220, PF221, PF222, PF223, PF224, and PF14, in combination to plasmid (pGL3), SCO and siRNA with and without MNPs^38^ were investigated. A schematic representation of the formation of the nanocomplexes is given in Fig. 1. MNPs were added to the solution of ONs followed by the addition of CPPs. Alternatively; the MNPs were first mixed with CPPs and followed by the addition of the oligonucleotides. However, the formed species in the later did not show any improvement of the gene expression. The syntheses of CPPs-pGL3-MNPs and CPPs-SCO-MNPs were performed in MQ water (pH = 5). The charge ratio of CPPs-pGL3^34^ and the molar ratios of CPPs-SCO^32^ and CPPs-siRNA as previously reported in our group^39^ were used under the optimum conditions.

**Figure 3:**
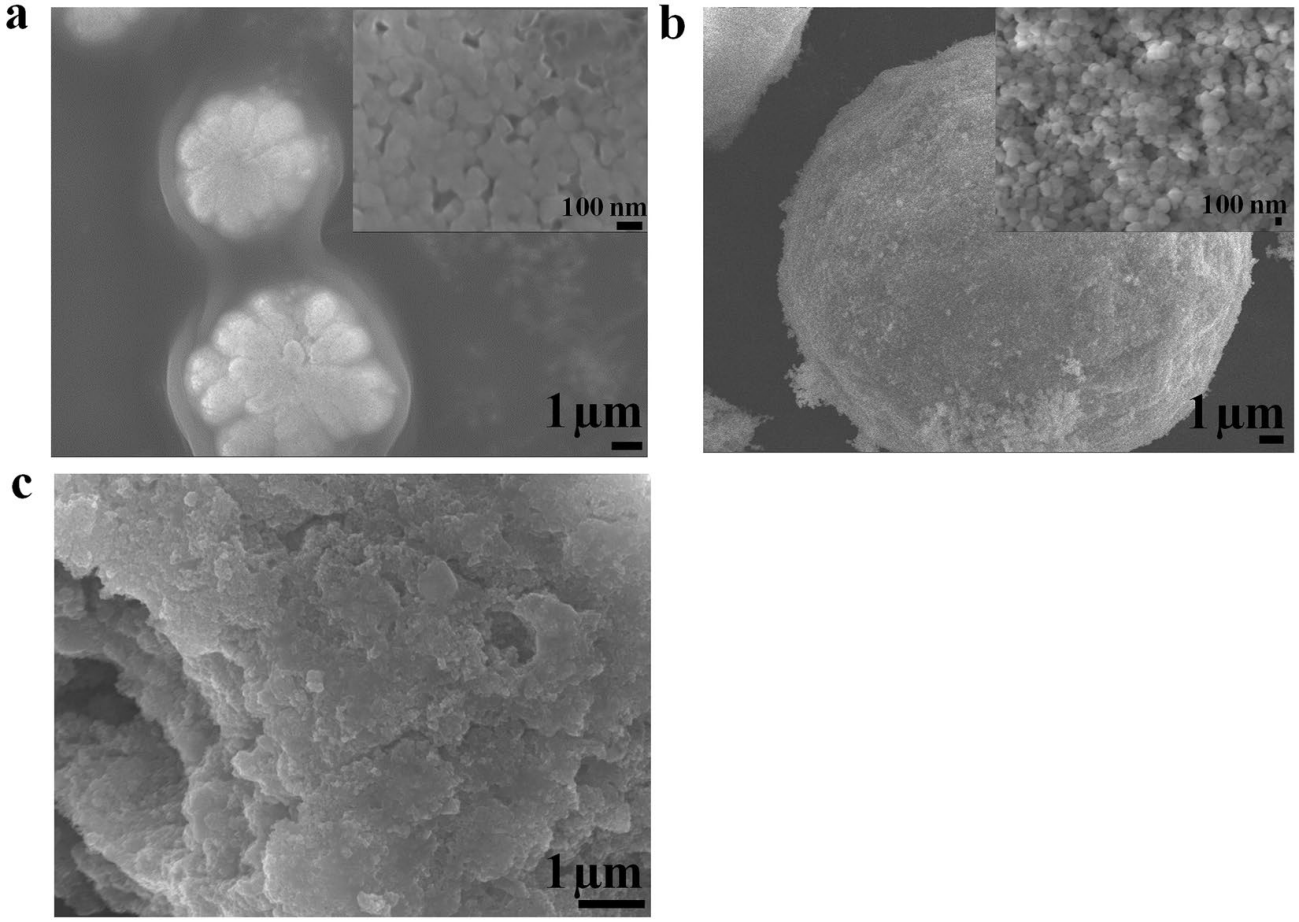
SEM images of (**a**) PF221-pGL3-MNPs, (**b**) PF220-SCO-MNPs and (**c**) PF14-siRNA-MNPs. The insets in (**a**) and (**b**) are enlarged areas showing the subunits of the nanocomplexes.

The morphology and chemical analysis of PF14 were performed using SEM and EDX, respectively (Figure S3). The particle sizes of PF14 were in the range of 100–500 nm. EDX confirmed the presence of C, N, O, Na and F. The presence of F and Na is due to the cleavage reagent (95% trifluoroacetic acid, 2.5% water, 2.5% triisopropylsilane) and the eluent reagent (acetonitrile/water containing 0.1% TFA) used for the protein cleavage and purification, respectively^32^. The morphologies of the complexes with and without the MNPs were studied using SEM (Figure S4). The complexes of PF14-pGL3, PF14-SCO, and PF14-siRNA are aggregation of the subunits (Figure S4(a–c)), and the MNPs are distributed in the complexes of CPPs-ONs (Figure S4(d–f)). PF221-pGL3-MNPs, PF220-SCO-MNPs, and PF14-siRNA-MNPs (Fig. 3) have well-defined morphology compared to the other CPPs-ONs-MNPs (Figure S5). SEM images of PF221-pGL3-MNPs show subunits of the nanocomplexes with sizes less than 100 nm (Fig. 3a). The morphology and the size of the nanocomplexes varied based on the chemical composition and the length of the CPPs. The well-defined morphologies of PF221-pGL3-MNPs and PF220-SCO-MNPs are presumably due to self-assembly of the three components (CPPs, gene therapeutic agent, and MNPs) via noncovalent electrostatic interactions. Different CPPs have different surface charge distributions and therefore interfere differently with MNPs and the gene therapeutic agents (pGL3, SCO, and siRNA). These interactions affect and alter the physicochemical properties of the nanocomplexes and eventually the biological activity.

The chemical analysis of PF14-siRNA-MNPs using EDX confirmed the presence of the ternary components (Figures S6–S10). Particle aggregations with a wide size distribution were observed in PF14-siRNA (0.3–6 μm, Figure S7a). The presence of siRNA and PF14 was confirmed by EDX (Figure S7b). The magnetic nanoparticles were distributed within the siRNA, as confirmed by SEM and EDX analysis (Figure S8). A variation of the Fe:P ratios was also observed, indicating the content of the MNPs varied from particle to particle (Figure S9). These variations may take place during the complex formation or sample drying. NaCl and NaF crystals were observed nearby the dried PF14-pGL3 complexes, which were confirmed using EDS for the sample PF14-pGL3 without MNPs (Figure S10). This indicates that Na^+^, Cl^−^ and F^−^ ions were pushed out of the complexes during the drying process.

**Figure 4:**
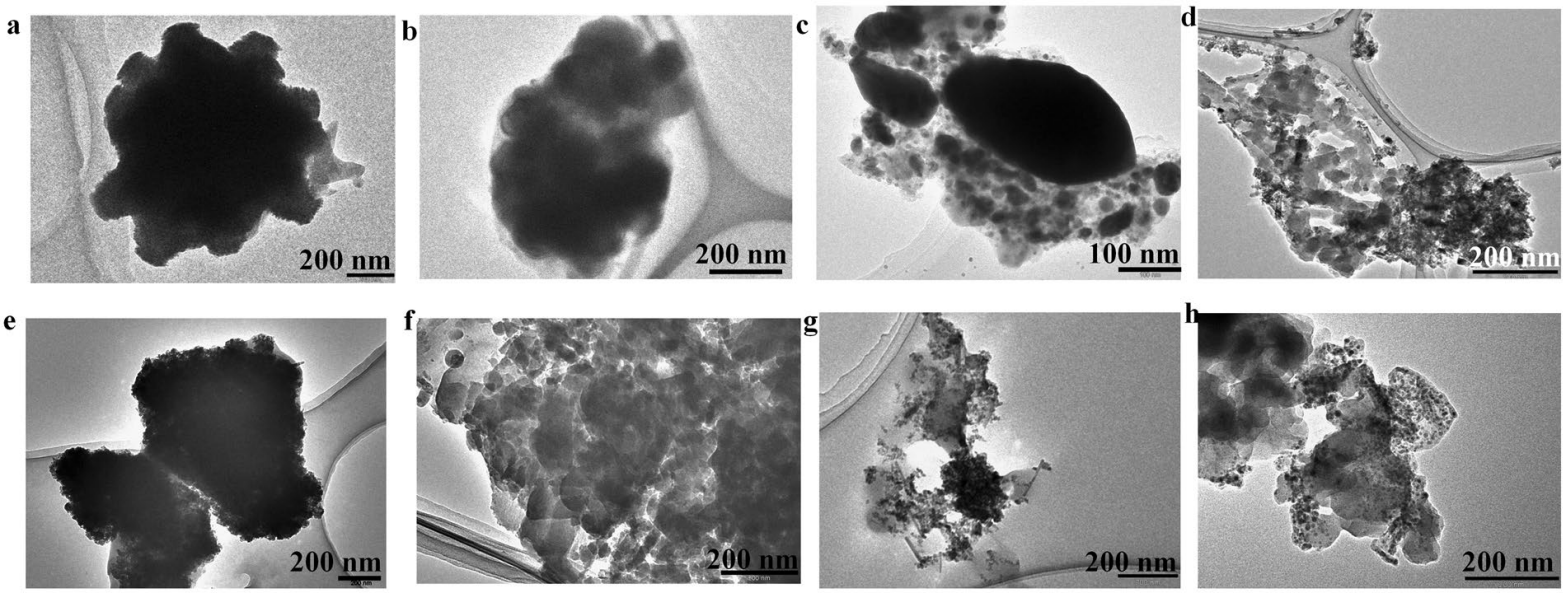
TEM images of (**a**) PF220-pGL3-MNPs, (**b**) PF221-pGL3-MNPs, (**c**) PF14-pGL3-MNPs), (**d**) PF220-SCO-MNPs, (**e**) PF221-SCO-MNPs, (**f**) PF14-SCO-MNPs, and PF14-siRNA-MNPs for siRNA of 25 nM (**g**) and 50 nM (**h**).

The typical morphology and size of CPPs-pGL3-MNPs, CPPs-SCO-MNPs and PF14-siRNA-MNPs (CPPs; PF220, PF221, and PF14) are shown in the TEM images (Fig. 4). EDX analysis using TEM confirmed the presence of MNPs within the complexes of PF14-siRNA (Figure S7). It is hard to estimate the individual particle size of the ternary complexes. The MNPs are dispersed in the complexes of CPPs-pGL3, CPPs-SCO, and CPPs-siRNA. They are more aggregated in the complexes of CPPs-pGL3 and CPPs-SCO (Fig. 4(a–f)) compared to those in the complexes of PF14-siRNA (Fig. 4(g–h)).

The surface modifications of MNPs with the CPPs-pGL3, PF14-siRNA, and CPPs-SCO complexes are confirmed by the corresponding zeta potentials (Fig. 2c). The synthesized MNPs were positively charged, as indicated by their zeta potential (2.40 ± 0.06 mV). All the investigated materials displayed positive charges except for complexes containing PF14 for pGL3 and SCO (Fig. 2c) and at a high concentration of siRNA (50 nM, Figure S11). All the species that have zeta potential outside the range of −30 to 30 mV produced stable colloidal solutions. The zeta potentials of MNPs and CPPs-pGL3-MNPs (CPPs; PF220, PF221, and PF223) reveal that they have low stability and tend to aggregate. The zeta potential of nanoparticles usually affects the cell uptake. Generally speaking, positively charged particles have higher uptake compared to negatively charged particles due to the electrostatic interactions of CPP/cargo complexes with plasma membranes^40^. Our group previously showed that PF14-ONs complexes were negatively charged and their uptake took place by a receptor^41^. An excess of PF14 neutralized the negative charges of siRNA when the concentration of siRNA was low (25 nM) and the complexes with and without MNPs had positive net surface charges (Figure S11). On the other hand, the net charge of the complex is negative at a higher concentration of siRNA (50 nM, Figure S11).

To further confirm the interaction of ONs with MNPs, fluorescence measurements of labeled ONs with Alexa Fluor 568 (Alexa Fluor 568 oligonucleotide amine) were performed (Figure S12). The principle of the assay is based on the fact that when MNPs bind to Alexa Fluor 568 oligonucleotide amines, the fluorescence signals increase substantially (Figure S12). The increase of fluorescence signal is due to the replacement and release of Alexa Fluor 568 oligonucleotide amines after the complexes have bound to the MNPs. Thus, the increase in fluorescence signals reflects the ability of MNPs to interact with complexes containing plasmid and CPPs. The increases of fluorescence signals are significant for PF222 and PF224 compared to other CPPs. This assay confirms that the interaction between MNPs and peptides does not out-compete the oligo from the complex.

### WST-1 cytotoxicity assay

To investigate the toxicity of the CPPs-pGL3-MNPs (CPPs; PF220, PF221, PF222, PF223, PF224, and PF14), mitochondrial reductase activities were measured using a commercial WST-1 assay (Figure S13). MNPs show high biocompatibility (Figure S13). Furthermore, none of the new complexes shows significant toxicity after the incubation for 24 h (Figure S13). The data confirm that these complexes are biocompatible and show no significant toxicities to the investigated cells.

### Activity for gene therapy

**Figure 5:**
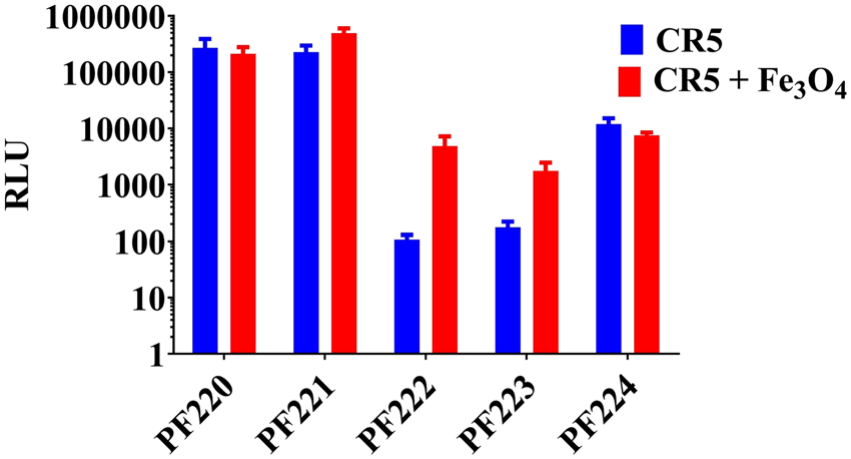
Transfection of the pGL3 plasmid in Hela cells for CPPs-pGL3 (CR5) and CPPs-pGL3-MNPs (CR5 + Fe_3_O_4_); while CPPs are PF220, PF221, PF222, PF223, and PF224 using a charge ratio of 5 (CR5), RLU refers to the relative light unit (RLU).

**Figure 6:**
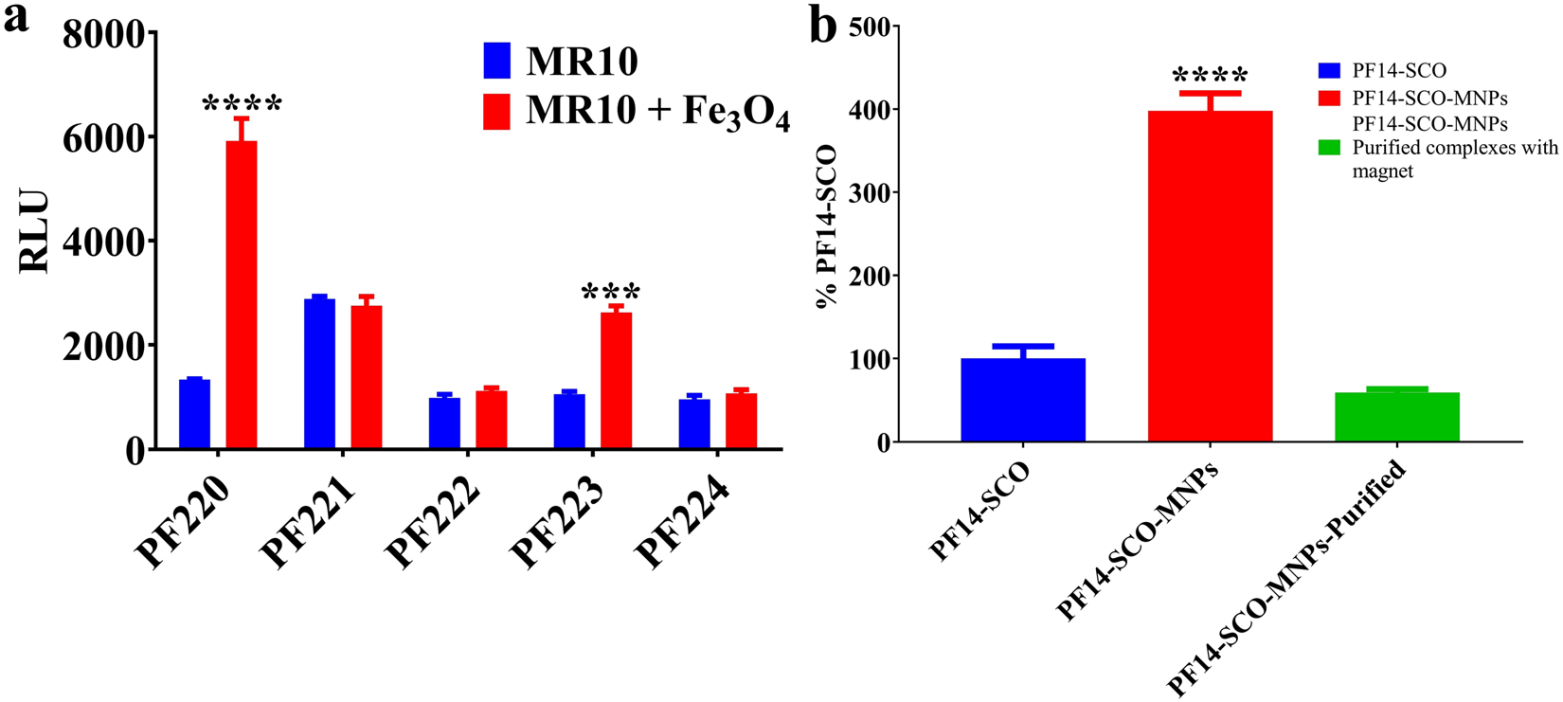
Splice-correction activity of (**a**) CPPs-SCO and CPPs-SCO-MNPs (CPPs of PF220, PF221, PF222, PF223 and PF224) and (**b**) PF14-SCO complexes and PF14-SCO-MNPs with and without purification.

**Figure 7:**
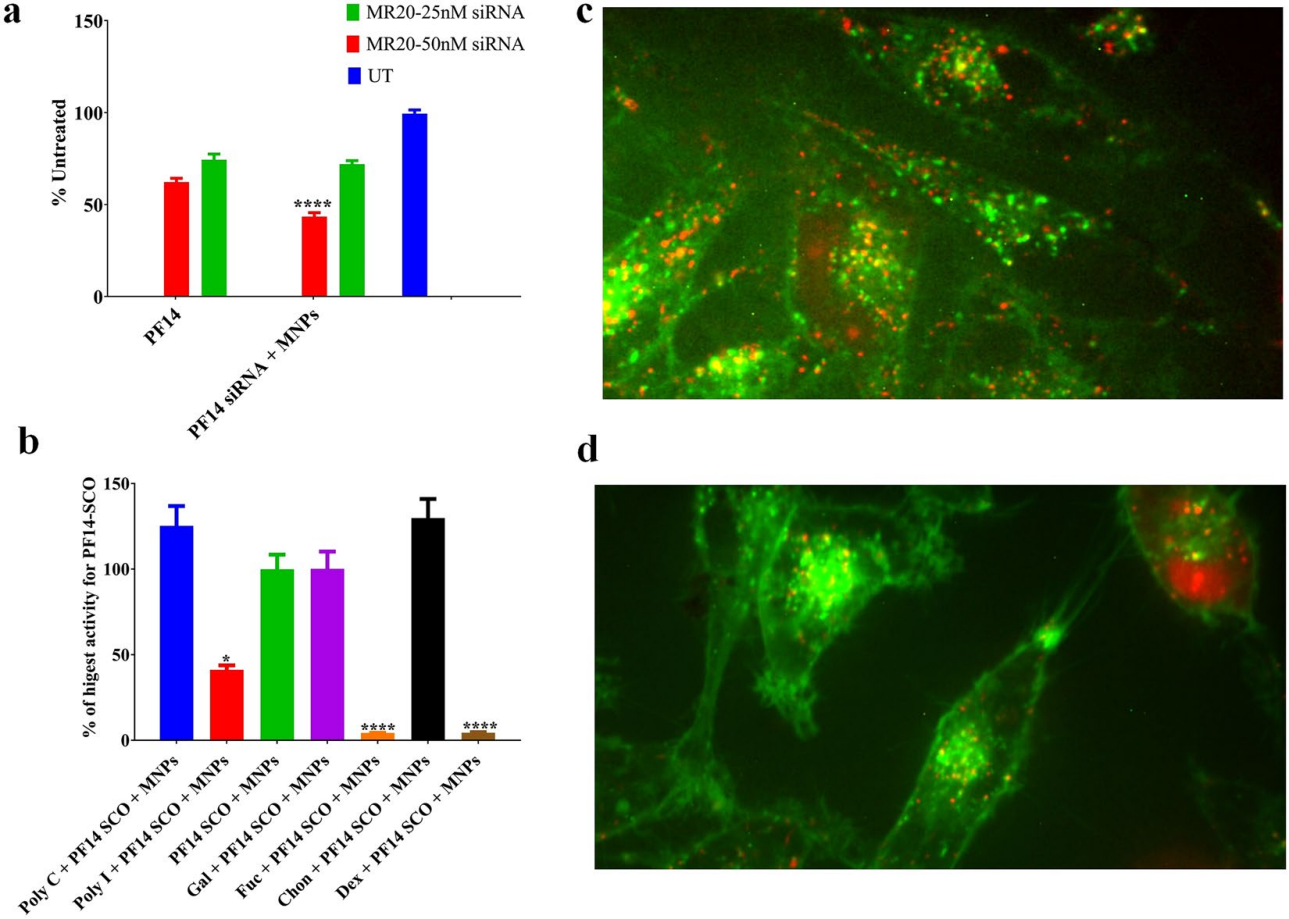
(**a**) PF14 and PF14-MNPs mediate efficient siRNA in U87 Luc cell-lines, (**b**) cell uptakes based on the mechanism of scavenger receptor class, (**c**,**d**) confocal microscopy images of Hela-705 cells incubated for 24 h with PF14-Alexa 568–705ASO (MR10, 100 nM ASO) complexes (in red) without (**c**) and with (**d**) MNPs. Cell-membranes are stained with Fast-dio stain (in green).

The efficiency of the prepared complexes for gene therapy was investigated. The stearylated form of the cell penetrating peptides (CPPs) was used to increase the uptake efficacy of CPPs^42^. We investigated the complexes at the optimum charge ratio (CR 5) for CPPs-pGL3-MNPs (Fig. 5)^34^, at a molar ratio (MR 10) for CPPs-SCO-MNPs (Figs 6 and S14)^32^ and MR 20 for siRNA (Fig. 7a).

### Plasmid Activity of CPPs-pGL3-MNPs

Transfection of CPPs-pGL3-MNPs (CPPs; PF220, PF221, PF222, PF223, and PF224) in Hela cells was investigated (Fig. 5). Hela cells were cultured for 24 h before treatment, and then CPPs-pGL3-MNPs with a charge ratio of 1:5 was added. Luminescence intensities were measured after the treatment. Statistical analysis using two-way ANOVA test of CPPs-pGL3-MNPs (CPPs; PF220, PF221, PF222, PF223, PF224) shows no significant improvements for PF220, or PF224 (Fig. 5). On the other hand, complexes of CPPs-pGL3-MNPs (CPPs; PF221, PF222, PF223) show higher activities (2–100 folds) compared to the corresponding CPPs-pGL3.

### Splice correction oligonucleotide (SCO) assay

SCOs are antisense oligonucleotides (ASO) varying from 15 to 25 bases in sequence^38^. In contrast to the normal antisense approach, SCO showed no induced RNase, which prevents the pre-mRNA target from being destroyed before it could be spliced^38^. The effects of molar ratio (20, 10, and 5), concentration (50, 100, and 200 nM) and the amount of MNPs (4 and 8 μL of MNPs (stock solution, 1 mg/mL)) were investigated (Figure S14). Data show that the efficiency decreased with the decrease of molar ratio. No noticeable change was recorded for MR 5 in the presence of MNPs. Interestingly; all the data confirm that the presence of MNPs improves the efficiency of the complex (PF14-SCO). A lower amount of MNPs showed higher efficiency compared to the high concentration of MNPs (Figure S14). Among the different reported CPPs, the efficiency was increased by 4 times for PF14-SCO when MNPs was incorporated (Fig. 6). It showed higher efficiency (transfect nearly 100% of the cell population) compared to a commercial lipid-based vector called Lipofectamine™ 2000 (LF2000)^32^. The complex of PF14-SCO-MNPs after separation using external magnets and redispersion in water showed a lower activity (Fig. 6). The low efficiency of the separated complex may be due to the aggregation of the complexes after the separation.

### Small interfering RNA (siRNA)

Knockdown specific target gene expression using siRNA with and without MNPs was also investigated. The delivery of siRNA causes degradation of the luciferase mRNA that leads to a decrease in the signal of luminescence^43^. Two different concentrations of siRNA (50 nM and 25 nM) were investigated (Fig. 7a). PF14 showed higher efficiency compared to other CPPs for siRNA^44^. At low concentration of siRNA (25 nM), PF14-siRNA with and without MNPs had the same effectiveness (Fig. 7a). However, PF14-siRNA-MNPs (50 nM) showed higher down-regulation of the target gene compared to PF14-siRNA with the same concentration (Fig. 7a). This observation indicates that MNPs offer a significant improvement for the gene transfection of siRNA. The high efficiency may be due to the concentration of siRNA or the high tendency to form stable suspension as shown by the zeta potential (Figure S11).

### Study of the cell uptake: Scavenger class A (SCARA) and confocal microscopy

So far, the exact route of the cell uptake of CPPs-ONs complexes is still vague^6, 45^. However, there are some reasonable pathways that could explain the cell uptakes. Among those endocytosis and energy-independent translocation^46^ and presence of scavenger class A (SCARA)^41^ are reasonable explanations for the cell uptakes of these complexes (Fig. 7b). SCARA is a pattern recognition receptor that can bind and endocytose acetylated low-density lipoprotein^41^. HeLa cells were incubated with inhibitor (Dextran sulfate (Dex), polyinosinic acid (Poly I) or fucoidin (Fuc)) or their respective control e.g. structurally similar compounds (Chondroitin sulfate (Chon), polycytidylic acid (Poly C) or galactose (Gal)) before addition of PF14-SCO-MNPs complexes (Fig. 7b). Chon, Poly C and Gal lack affinity to SCARA. The SCO activity mediated by PF14-MNPs is strongly inhibited using any inhibitor. The corresponding inhibitor controls show no significant inhibition of the SCO activity (Fig. 7b). Dextran sulfate and fucoidin (Fuc) inhibit SCO transfection mediated by PF14-SCO-MNPs by more than 90%. These observations confirm the high impact of scavenger receptor A in the uptake of PF14-SCO-MNPs^47^.

Oligonucleotides are often trapped in endosomes which have vesicular structures within the cell seen as punctate structures (Fig. 7c,d). The activity increases with the increase of the endosome escape rate of the nucleic acids. This can be achieved using delivery agents such as CPPs^27^ or CPPs-MNPs. The staining patterns of cells treated with MNPs containing PF14-Alexa 568–705ASO complexes showed little difference to cells treated with control complexes (without MNPs, Fig. 7c,d). Thus, the MNPs induced no changes of the cell uptake compared to the conventional CPPs-ONs complexes. However, they have higher transfection efficiency, indicating that a larger fraction of ONs can escape vesicular confinement. A previous study of intracellular trafficking of SPIO conjugated with transactivating transcriptional activator (TAT) peptides derived from HIV-1 proteins by electron tomography confirmed the internalization of this complex inside the cell^48^. However, this study relied on conjugation of the CPPs with the MNPs rather than complexation. Our data showed that the complexation of MNPs with CPPs-ONs have several advantages. The MNPs enhanced the nucleic acid activity (Fig. 6). PF14-SCO-MNPs shows 4 times higher efficiency compared to PF14-SCO complexes. The material efficiency may be increased further by activation using thermal energy or magnetic field. CPPs-siRNA-MNPs encapsulated in liposomes showed higher efficiency after activation using thermal and magnetic effects^49^. Thus, we believe that the effectiveness of these complexes can be further increased using activation via thermal, magnetic field or infrared laser. Our results confirm the recent report showing that inorganic nanoparticle based delivery had a higher efficiency than organic based platform^9^. The physio-chemical properties of MNPs improved the water-solubility and stability of the complexes^32^. The small particle size of MNPs offered high adsorption capacity for CPPs-ONs complexes.

### *In vivo* study

**Figure 8:**
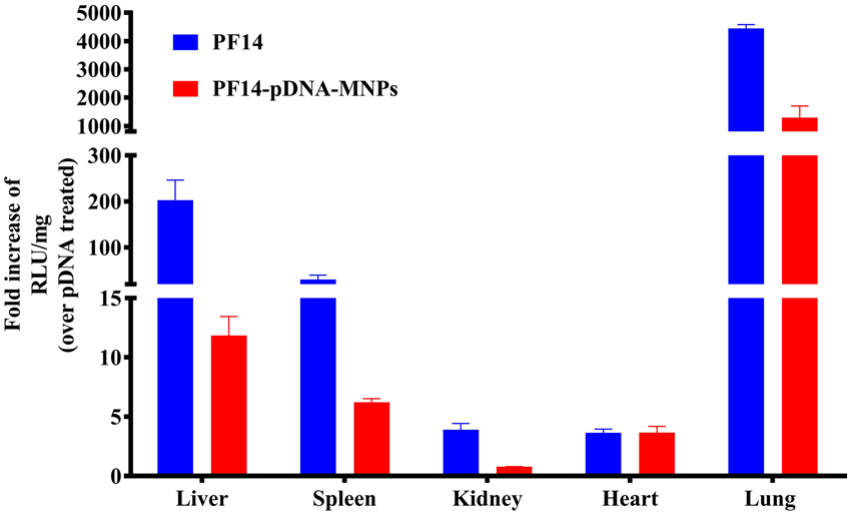
*In vivo* gene delivery efficacies of PF14 and PF14-pDNA-MNPs at N/P4 using pLuc. The data are represented as a fold increase of RLU/mg over the treatment with pDNA. N represents 2–5 repeated experiments.

The effects of the CPP-pDNA-MNPs were evaluated *in vivo*, using systemic administration of a single dose of pDNA (Fig. 8). We assessed the efficacy of PF14 because its potency *in vivo* has been shown before^50^. As shown in Fig. 8, the efficacy of PF14-pDNA-MNPs is comparable to that of the PF14, providing efficient transfection of the lungs and spleen, although the liver transfection decreased by 18-fold in the presence of MNPs. However, the MNPs formulation allows targeting of the particles using a magnetic field, which would be assessed in the future studies^19^.

## Conclusions

A new nanoplatform for gene transfer has been proposed. Hybrid gene transfer systems using MNPs and CPPs have been demonstrated for transfection of plasmid (pGL3), SCO and siRNA. A higher performance with the presence of MNPs was observed compared to traditional CPPs-pGL3, CPPs-SCO and CPPs-siRNA complexes. PF14-SCO-MNPs showed a higher activity (up to 4 times) compared to the PF14-SCO complex. The current results contribute to the strategies of gene transfer with magnetic nanoparticles.

## Electronic supplementary material

Supporting information
