# Supplementary material for: Magnetic Nanoparticle Assisted Self-assembly of Cell Penetrating Peptides-Oligonucleotides Complexes for Gene Delivery
Source: Sci Rep. 2017 Aug 22;7:9159. doi: 10.1038/s41598-017-09803-z (PMC5567346; doi:10.1038/s41598-017-09803-z)
Supplement: Supplementary file 1 — Supporting information [file 41598_2017_9803_MOESM1_ESM.pdf]

Magnetic Nanoparticle Assisted Self-assembly of Cell Penetrating Peptides-Oligonucleotides Complexes for Gene Delivery

Moataz Dowaidar*§1, Hani Nasser Abdelhamid§2, Mattias Hällbrink[1], Krista Freimann[3], Kaido Kurrikoff[3], Xiaodong Zou*[2], Ülo Langel*[1]

[1]Department of Neurochemistry, Stockholm University, Svante Arrhenius väg 16B, Stockholm, SE-10691 Sweden

[2]Department of Materials and Environmental Chemistry, Stockholm University, Svante Arrhenius väg 16C, Stockholm, SE-106 91, Sweden

[3]Laboratory of Molecular Biotechnology, Institute of Technology, University of Tartu, Nooruse, 50411 Tartu, Estonia

*Correspondence may be addressed to M. Dowaidar. Tel: +4672019203, Fax: +46816137, Email: moataz@neurochem.su.se; Xiaodong Zou. Tel: +468162389; Fax: +46 8152187; Email: xzou@mmk.su.se; Ülo Langel. Tel: +46-8-161 793; Fax: +46816137; Email: ulo@neurochem.su.se

§ Those authors share the first author and have equal contributions

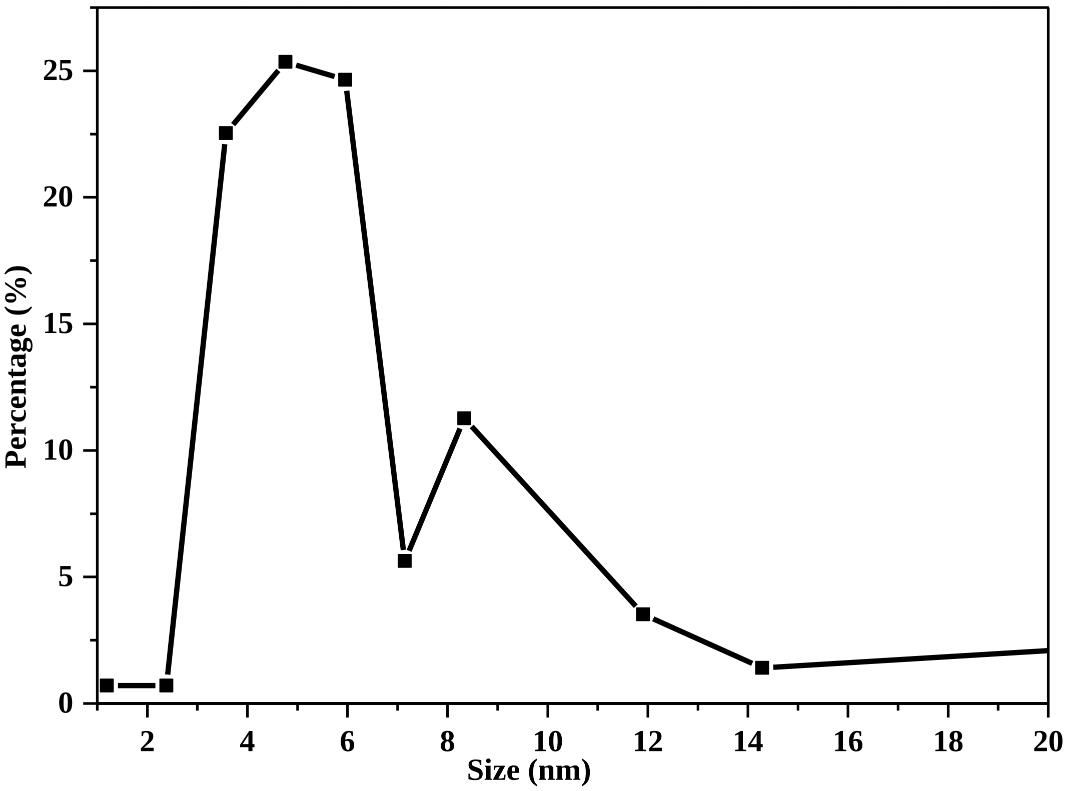

Figure S1 Particle size distribution of the magnetic nanoparticles (MNPs).

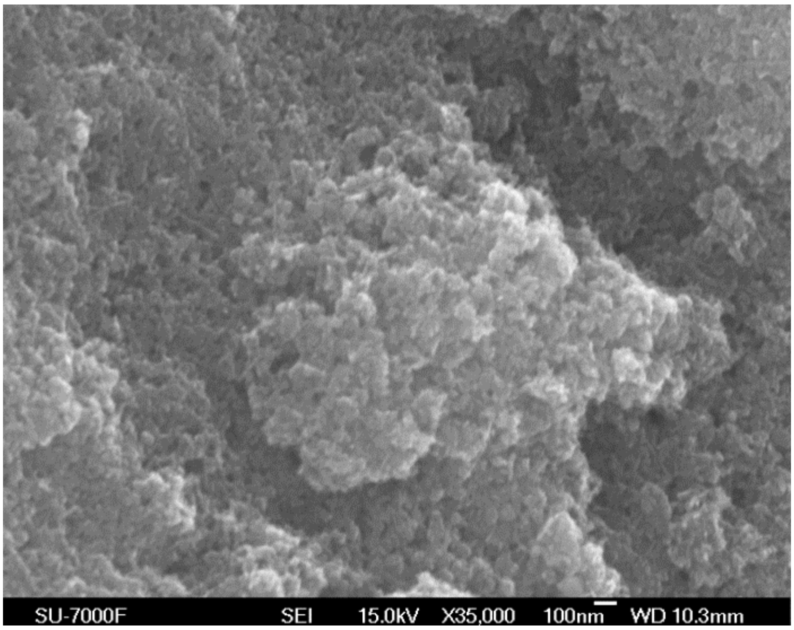

Figure S2 SEM image of the MNPs.

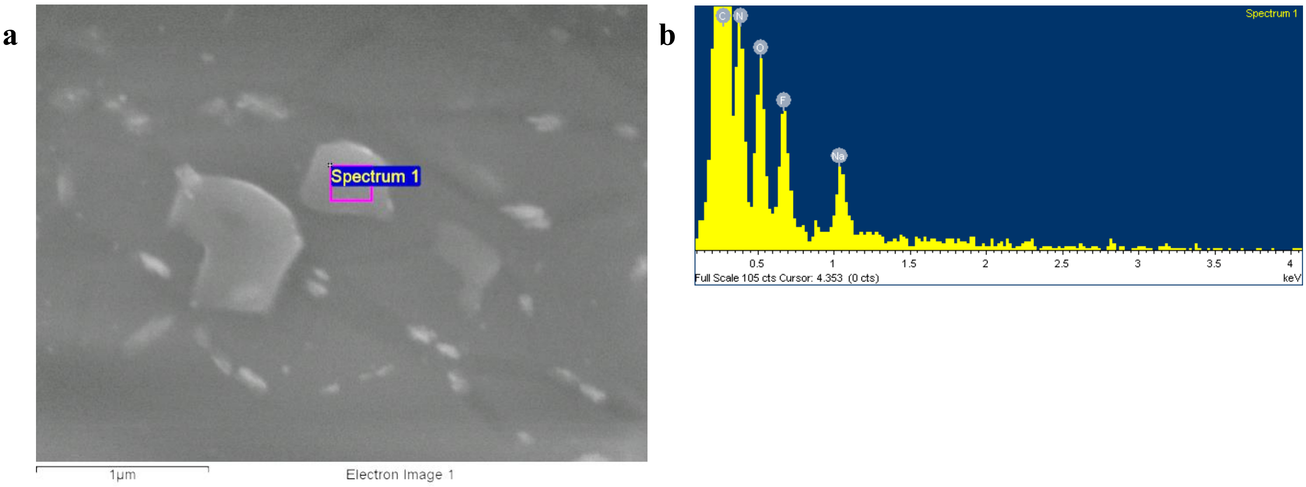

Figure S3 a) SEM image and b) EDX spectrum of PF14.

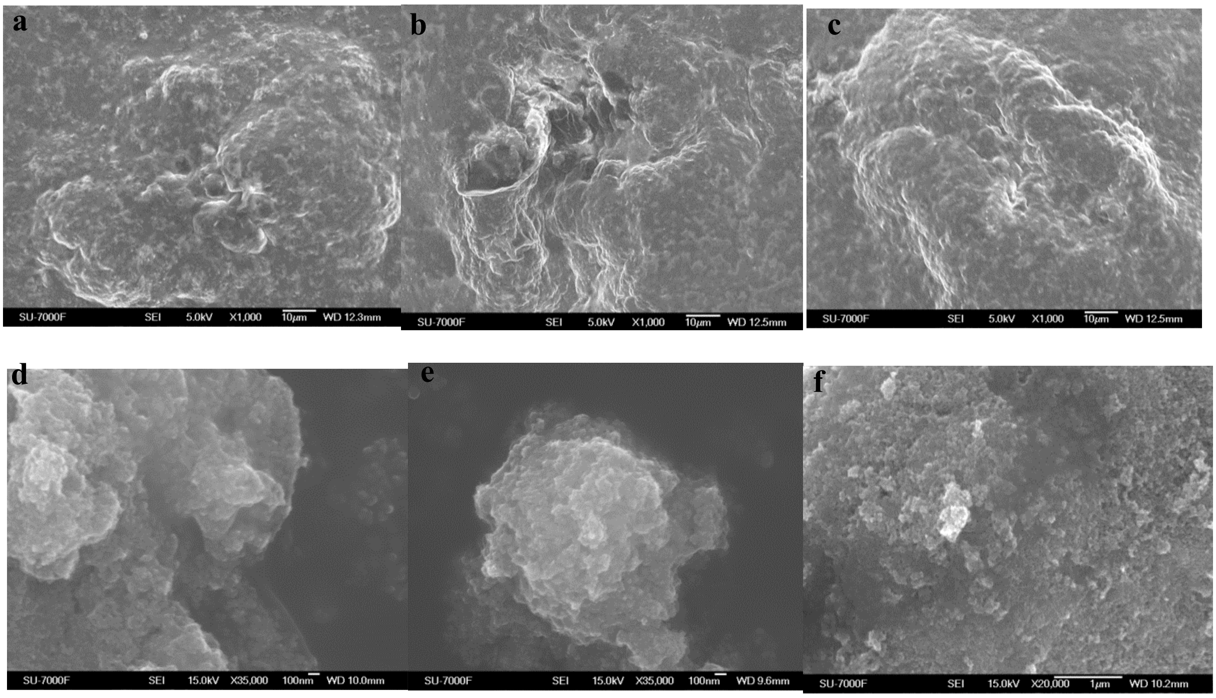

Figure S4 SEM images of a) PF14-pGL3, b) PF14-SCO, c) PF14-siRNA, d) PF14-pGL3-MNPs, e) PF14-SCO-MNPs, f) PF14-siRNA-MNPs.

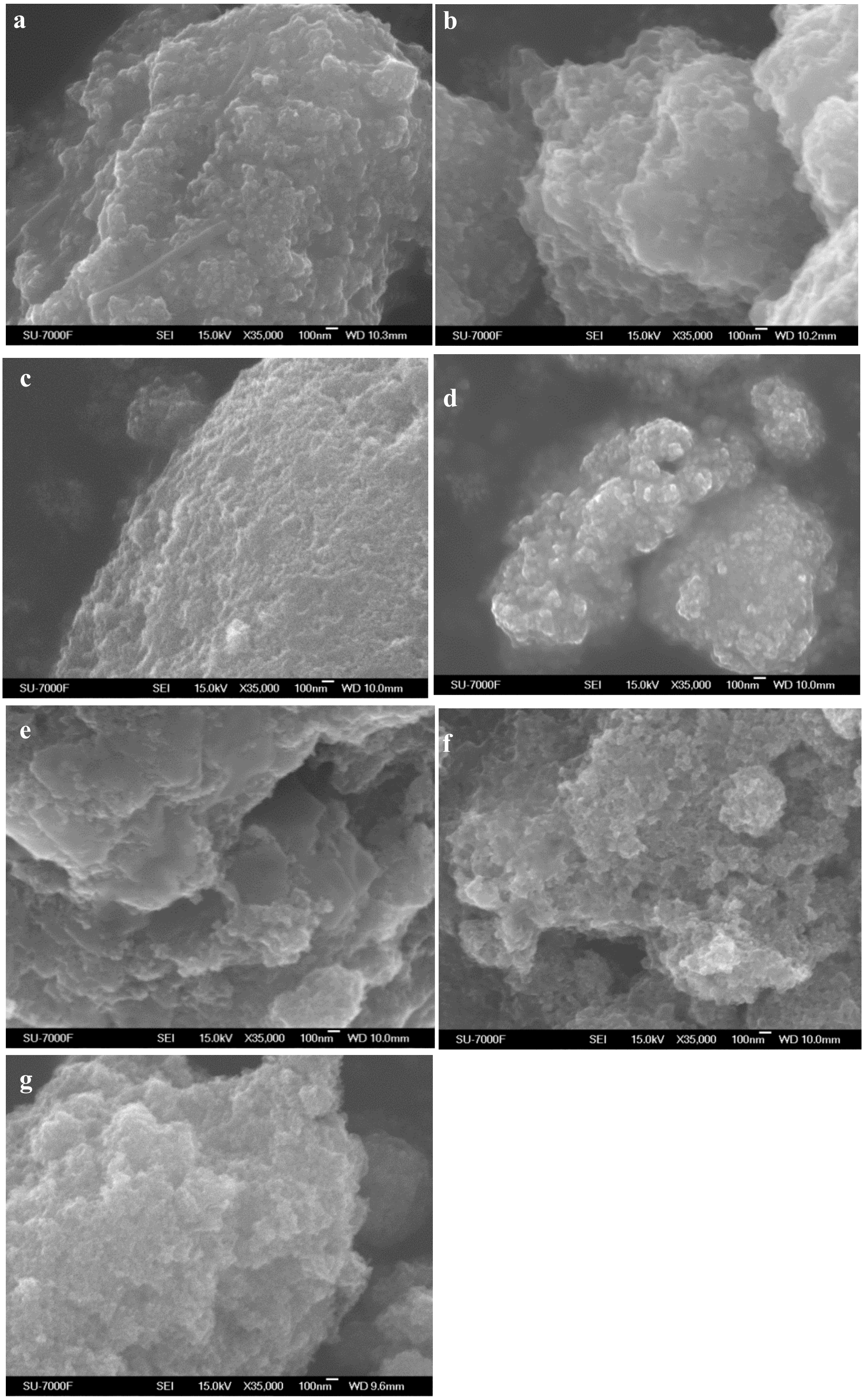

Figure S5 SEM images of CPPs-pGL3-MNPs (where CPPs for a) PF220, b) PF222, c) PF223, d) PF224 and, and e-g) CPPs-SCO-MNPs (where CPPs for e) PF221, f) PF221, and g) PF14).

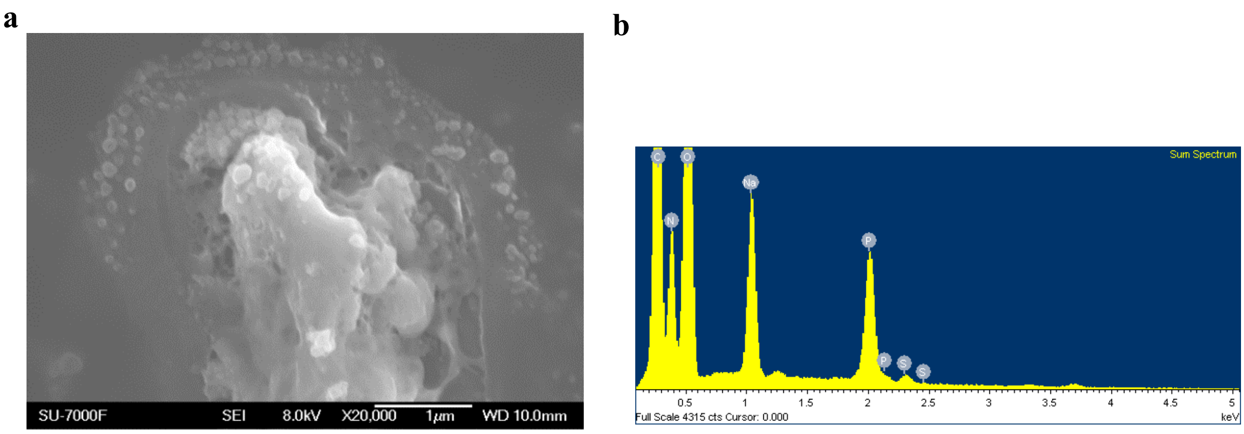

Figure S6 a) SEM images of siRNA and b) EDX analysis.

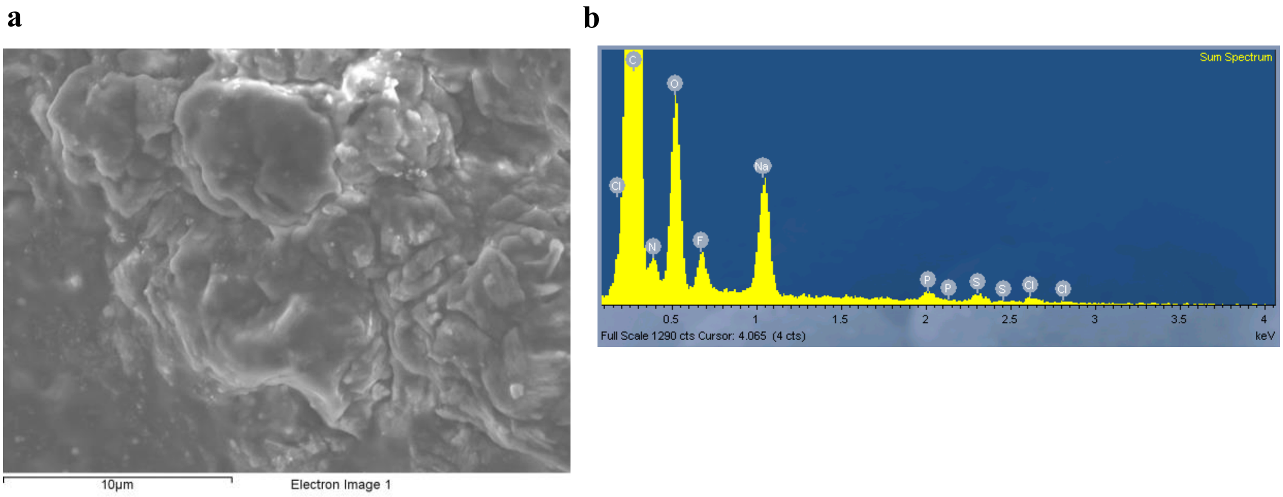

Figure S7 a) SEM images of PF14-siRNA and b) EDX analysis.

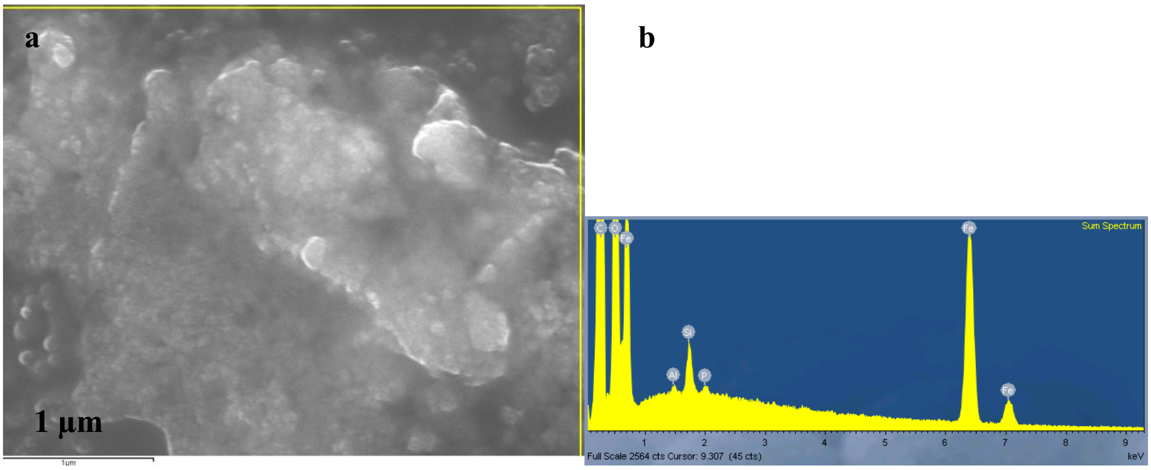

Figure S8 a) SEM images of siRNA-MNPs and b) EDX analysis. The presence of Al or Si is due to the SEM holder.

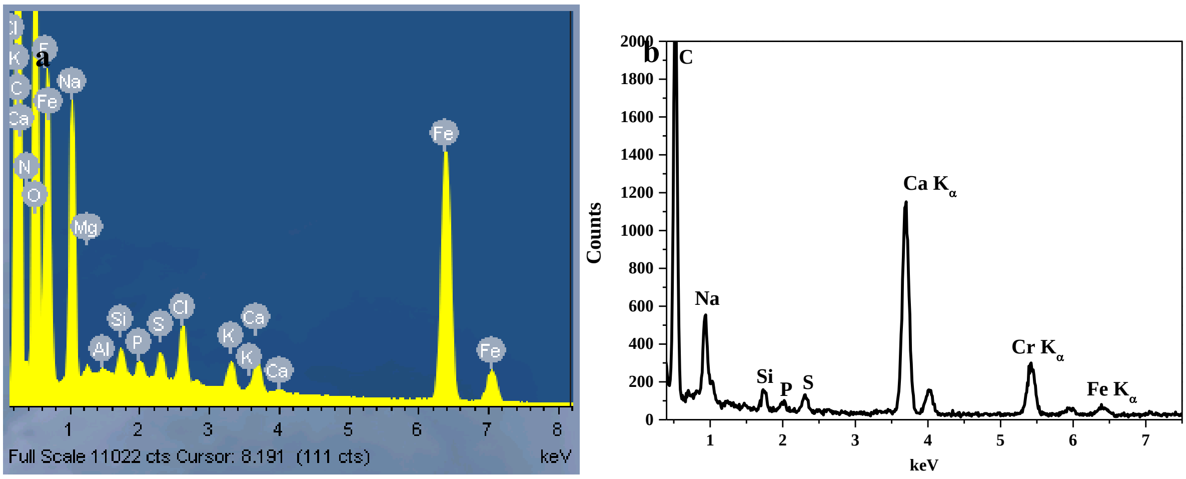

Figure S9 EDX analysis of PF14-siRNA-MNPs using a) SEM and b) TEM.

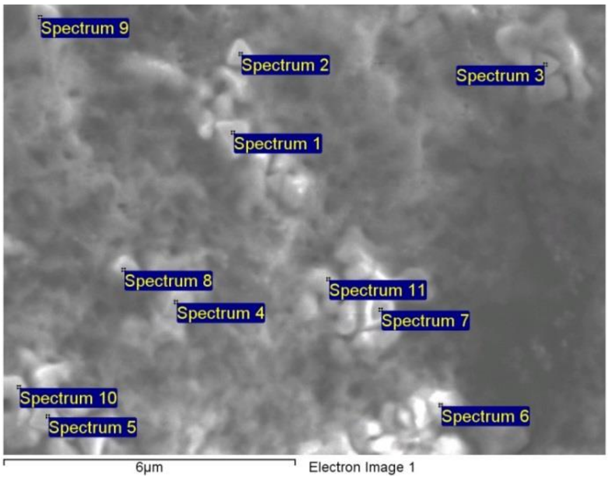

Spectrum	C	O	F	Na	Cl	Total
Spectrum 1	1.9	6.5	51.0	38.9	1.7	100
Spectrum 2	6.6	4.4	48.3	40.5	0.2	100
Spectrum 3	1.8	5.6	46.3	44.6	1.7	100
Spectrum 4	3.8	6.6	50.8	38.8	0	100
Spectrum 5	6.2	5.0	38.0	37.9	12.9	100
Spectrum 6	16.0	7.3	0.1	31.4	45.2	100
Spectrum 7	3.9	5.0	45.7	45.2	0.2	100
Spectrum 8	18.3	10.5	5.00	28.2	38.00	100
Spectrum 9	5.2	5.4	47.0	41.0	1.4	100
Spectrum 10	18.1	6.3	3.4	23.1	49.1	100
Spectrum 11	36.7	20.0	7.1	21.4	14.8	100

Figure S10 SEM image and EDX analysis of the crystal that observed surround the complex of PF14-pGL3.

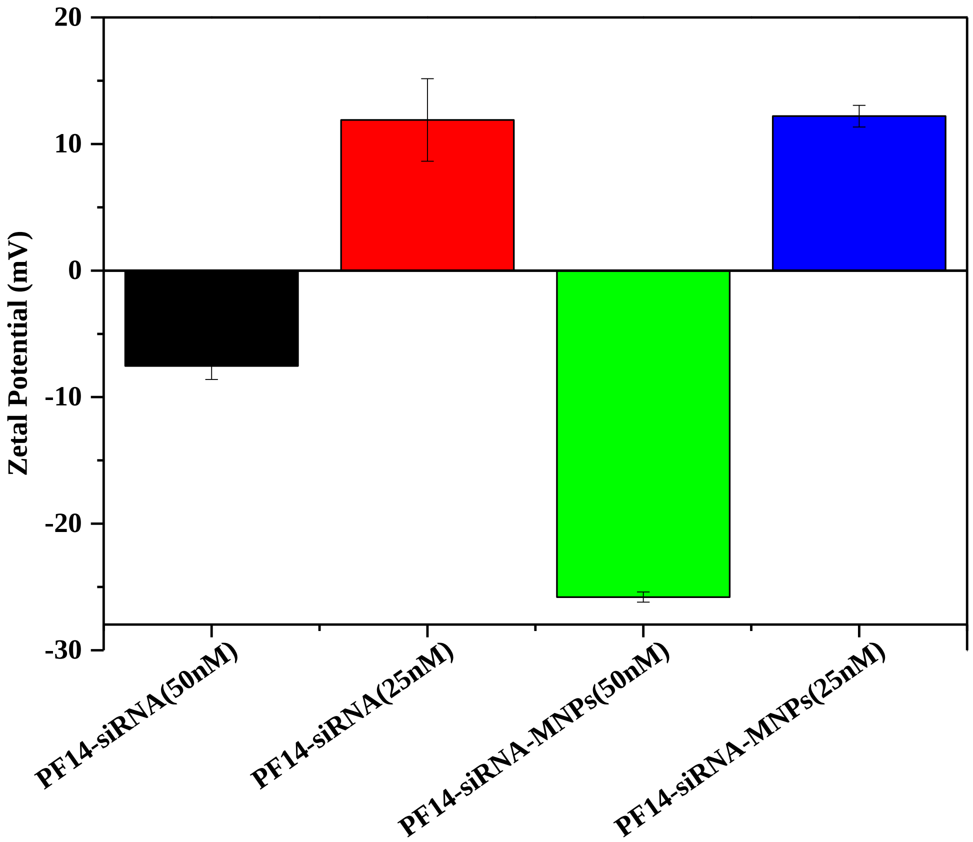

Figure S11 Zeta potentials of PF14-siRNA with and without MNPs ([siRNA] is 50 nM and 25 nM).

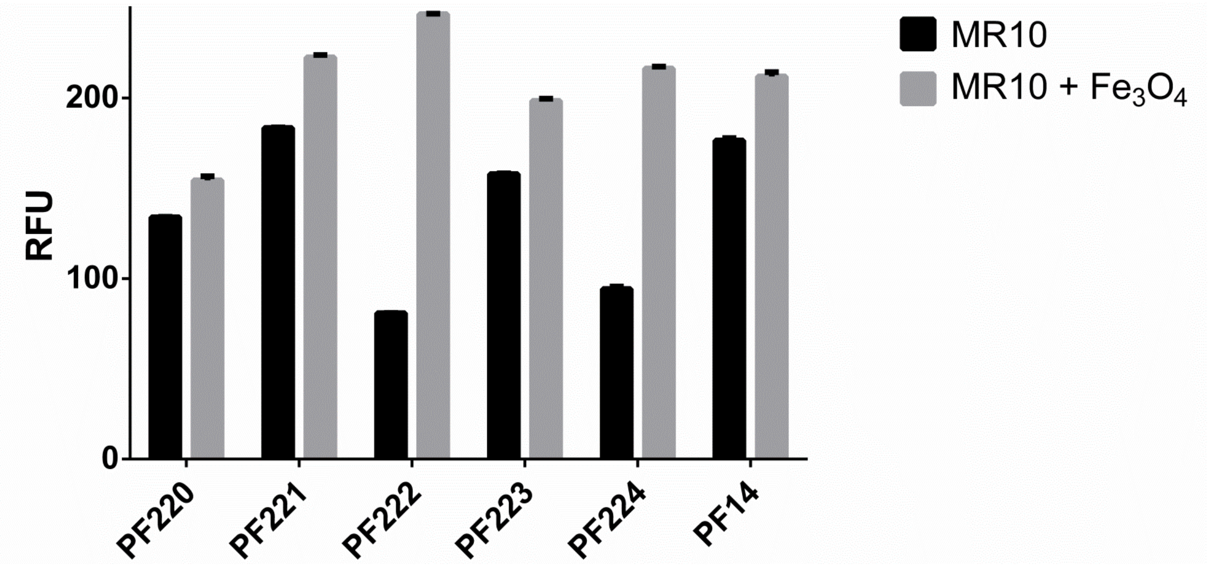

Figure S12 Fluorescence signals of Alexa Fluor 568 oligonucleotide amines with and without the presence of MNPs.

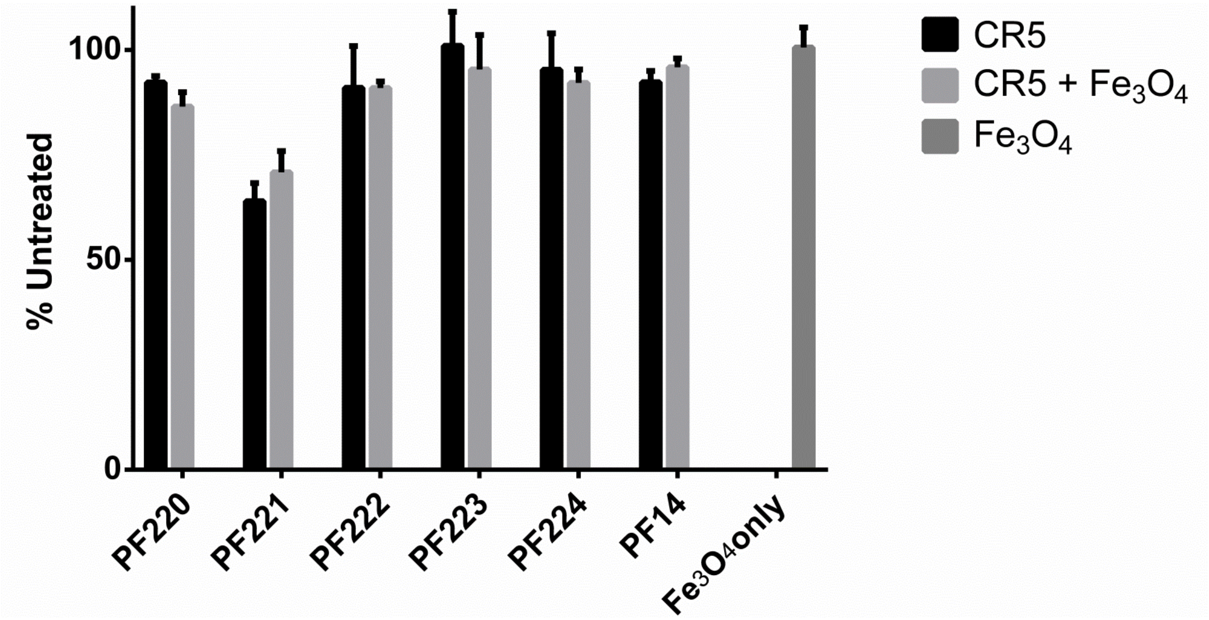

Figure S13 WST-1 assay for the biocompatibility of the prepared materials.

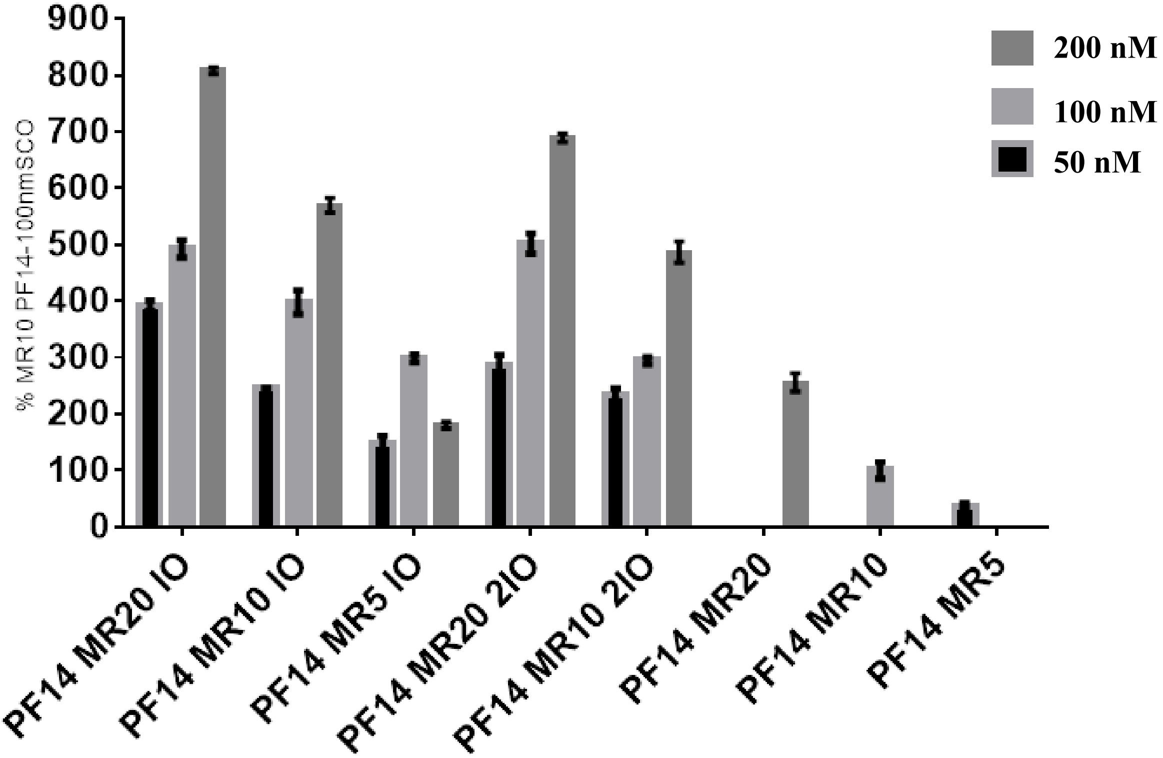

Figure S14 Effects of molar ratio (PF14:SCO), concentrations of SCO (50, 100 and 200 nM) and MNPs (Fe_3O_4, 4 μL and 8 μL of stock solution, 1 mg/mL).
